# Foot-and-Mouth Disease (FMD) Virus 3C Protease Mutant L127P: Implications for FMD Vaccine Development

**DOI:** 10.1128/JVI.00924-17

**Published:** 2017-10-27

**Authors:** Michael Puckette, Benjamin A. Clark, Justin D. Smith, Traci Turecek, Erica Martel, Lindsay Gabbert, Melia Pisano, William Hurtle, Juan M. Pacheco, José Barrera, John G. Neilan, Max Rasmussen

**Affiliations:** a U.S. Department of Homeland Security Science and Technology Directorate, Plum Island Animal Disease Center, Greenport, New York, USA; b Leidos, Inc., Plum Island Animal Disease Center, Greenport, New York, USA; c Oak Ridge Institute for Science and Education, Plum Island Animal Disease Center Research Participation Program, Oak Ridge, Tennessee, USA; University of California, Irvine

**Keywords:** 3C protease, StopGo translation, Escherichia coli, Gaussia luciferase, VLP, adenoviruses, foot-and-mouth disease virus, *in vivo*, *in vivo* expression technology, translational interrupter, vaccines, virus-like particles

## Abstract

The foot-and-mouth disease virus (FMDV) afflicts livestock in more than 80 countries, limiting food production and global trade. Production of foot-and-mouth disease (FMD) vaccines requires cytosolic expression of the FMDV 3C protease to cleave the P1 polyprotein into mature capsid proteins, but the FMDV 3C protease is toxic to host cells. To identify less-toxic isoforms of the FMDV 3C protease, we screened 3C mutants for increased transgene output in comparison to wild-type 3C using a Gaussia luciferase reporter system. The novel point mutation 3C(L127P) increased yields of recombinant FMDV subunit proteins in mammalian and bacterial cells expressing P1-3C transgenes and retained the ability to process P1 polyproteins from multiple FMDV serotypes. The 3C(L127P) mutant produced crystalline arrays of FMDV-like particles in mammalian and bacterial cells, potentially providing a practical method of rapid, inexpensive FMD vaccine production in bacteria.

**IMPORTANCE** The mutant FMDV 3C protease L127P significantly increased yields of recombinant FMDV subunit antigens and produced virus-like particles in mammalian and bacterial cells. The L127P mutation represents a novel advancement for economical FMD vaccine production.

## INTRODUCTION

Foot-and-mouth disease virus (FMDV) is a worldwide threat to food security, production, and trade, with approximately 2.35 billion doses of foot-and-mouth disease (FMD) vaccines administered to livestock annually (1). Efficacious FMD vaccines require production of intact, assembled FMDV capsids to induce a protective immune response. Conventional FMD vaccines are produced by chemical inactivation of purified, virulent FMDV virions, but this production method is inherently risky with respect to accidental release of FMDV. Current U.S. law (21 U.S.C. 113A) prohibits introduction of live FMDV into the U.S. mainland for any purpose, which precludes manufacture of inactivated FMDV vaccines within the United States. Recombinant subunit vaccines are a safer alternative to traditional FMDV vaccines because they express only the FMDV proteins required for assembly of empty FMDV capsids, or virus-like particles (VLPs) (2–5). Molecular recombinant subunit vaccines containing only the FMDV genes needed to induce immunity have been developed and recently licensed (2–4).

Both inactivated and subunit FMD vaccines require cytosolic expression of FMDV 3C protease to process the FMDV P1 polyprotein into individual viral capsid proteins (VP0, VP3, VP1) (4, 6–10). However, wild-type FMDV 3C protease [3C(wt)] also cleaves mammalian host cell proteins, including histone H3 (11), eukaryotic initiation factor 4 subunit AI (eIF4AI) (12, 13), eIF4GI (12, 14), 68 kDa Src-associated substrate during mitosis (SAM68) (15), and nuclear transcription factor kappa B essential modulator (NEMO) (16), and continuous expression of FMDV 3C is toxic in bacteria (17–21). Consequently, use of wild-type 3C protease to produce recombinant FMD subunit vaccines limits expression platform options and antigen yields due to negative effects on host cells.

We sought to identify FMDV 3C mutations that increased yields of recombinant antigens in transfected cells compared to wild-type 3C. To screen for increased yields, FMDV 3C protease variants were linked with Gaussia princeps luciferase (GLuc) expression and separated by the FMDV translational interrupter sequence (Δ1D2A on a bicistronic gene cassette) (22). Mammalian cells expressing the 3C(L127P) mutation had significantly increased recombinant protein production compared to cells expressing wild-type FMDV 3C protease. The 3C(L127P) mutant was further analyzed for the ability to process the P1 polypeptide from multiple FMDV serotypes, for its effect on host proteins, and for the production of VLPs in both mammalian and bacterial cells. Last, we show that cattle immunized with an adenovirus (Ad)-vectored FMDV vaccine using the 3C(L127P) protease are protected from clinical FMD after homologous challenge.

## RESULTS

### Effect of 3C mutations on transgene expression.

**FIG 1:**
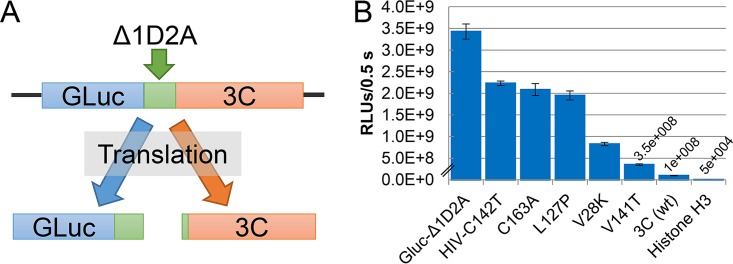
(A) Layout of the bicistronic GLuc-3C reporter assay used to evaluate 3C mutants and protein products. (B) Luciferase readings from culture media of HEK293-T cells transfected with GLuc-3C constructs (data represent an average of 6 samples/3C construct ± standard deviations).

The FMDV 3C protease is highly conserved among all seven FMDV major serotypes (23). We evaluated five 3C variants for their effect on recombinant protein (GLuc) yields compared to 3C(wt) using a bicistronic vector (chimera of Gaussia luciferase and the FMDV 3C protease [GLuc-3C]) (Fig. 1A). The variants included three novel 3C mutations (V28K, L127P, and V141T), the C163A mutant that inactivates 3C proteolytic activity (24), and a construct that downregulates 3C activity by combination with an HIV frameshift sequence (HIV-C142T) to reduce 3C translation to approximately 5% of normal expression levels (9).

All five 3C variants significantly increased GLuc yields over wild-type 3C yields in mammalian cells (*P* < 0.001) (Fig. 1B). Mutants 3C(C163A), 3C(HIV-C142T), and 3C(L127P) had the highest increases (Fig. 1B) and produced enhanced GLuc levels by three different mechanisms: an inactive protease (C163A), lower protease levels (HIV-C142T), and an uncharacterized mechanism (L127P). The 3C(L127P) and 3C(C163A) mutants were both expressed at high concentrations, and they were not statistically different from each other (*P* = 0.08), suggesting that the L127P mutation alters proteolytic functions (Fig. 1B). A *post hoc* analysis showed that GLuc yields were higher with the 3C(L127P) mutant than with either the 3C(V28K) mutant or the 3C(V141T) mutant (*P* < 0.001 to 0.04).

### FMDV P1 polyprotein processing by 3C(L127P) in mammalian cells.

**FIG 2:**
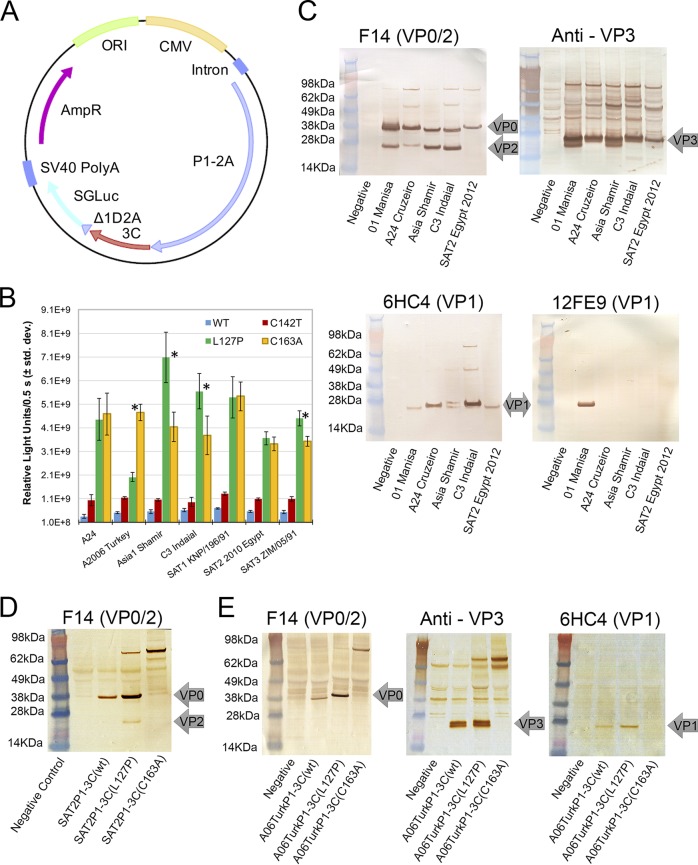
(A) Plasmid construct used for expressing FMDV P1-3C-SGLuc to evaluate P1 processing, transgene expression, and VLP formation. CMV, cytomegalovirus; SV40, simian virus 40. (B) Luciferase readings from culture media of mammalian cells expressing FMDV P1-3C-SGLuc constructs containing the 3C wild type (WT) or C142T, L127P, or C163A mutants (data represent an average of 7 samples/3C construct ± standard deviations [std. dev.]). *, *P* < 0.001 (for comparisons of luciferase RLU/0.5 s for L127P and C163A by a two-tailed *t* test). There was significant difference between the RLU/0.5 s values from L127P and those from both the wt and the C142T mutant for each serotype by a two-tailed *t* test (*P* < 0.001). (C) Western blots of transfected HEK293-T cell lysates showing P1 products from five FMDV serotypes processed by 3C(L127P). (D) FMDV SAT2 P1-expressing cells analyzed using an anti-VP0/VP2 monoclonal antibody. (E) FMDV A2006 Turkey P1-expressing cells analyzed using anti-VP0/VP2, anti-VP3, and anti-VP1 monoclonal antibodies.

To characterize FMDV P1 polyprotein processing by 3C variants, plasmids were made encoding transgene cassettes of a FMDV P1 polyprotein, a FMDV 3C protease wt or mutant, and the Δ1D2A-SGLuc reporter in a single open reading frame (Fig. 2A) (22). P1 polyproteins from six FMDV serotypes were combined with one of four 3C sequences (wild-type, C142T, L127P, or C163A) and processing in HEK293-T cells analyzed by Western blot with lysate loading equalized based on the luciferase data shown in Fig. 2B. SGLuc levels in cell culture media were significantly (*P* < 0.001 [one-tailed *t* test]) higher for the 3C(L127P) mutant than for 3C(wt) and 3C(C142T) strains, indicating increased recombinant protein yields from cells expressing 3C(L127P) (Fig. 2B), in agreement with results from the bicistronic system.

Western blot analysis that showed 3C(L127P) processed the P1 polyprotein from FMDV serotypes O, A, Asia1, C3, and SAT2 into the individual FMDV capsid proteins VP0, VP1, and VP3 (Fig. 2C to E). Detection of VP2 in the cell lysates from serotype strains SAT2, O1, A24, Asia1, and C3 demonstrated separation of VP0 into VP2 and VP4, which indicates the occurrence of capsid assembly (25) (Fig. 2C and D). However, differential levels of antibody binding affinity among serotypes resulted in different band intensities among samples. For example, to visualize the presence of SAT2 VP2, additional lysate needed to be loaded (Fig. 2D). While processing of A2006 Turkey P1 into VP0, VP3, and VP1 was seen by Western blotting, VP2 was not detected despite testing increased amounts of lysate (Fig. 2E). Interestingly, polyclonal antibody detection of VP3 resulted in the observation of a doublet of VP3 bands for serotypes O1 Manisa, Asia Shamir, and A2006 Turkey but no such result was observed for A24, C3 Indaial, or SAT2 Egypt 2012 (Fig. 2C and E). Processing of SAT1 and SAT3 P1 polyproteins was not assessed by Western blotting due to a lack of reactive monoclonal antibodies for those serotypes.

### VLP production in mammalian cells.

**FIG 3:**
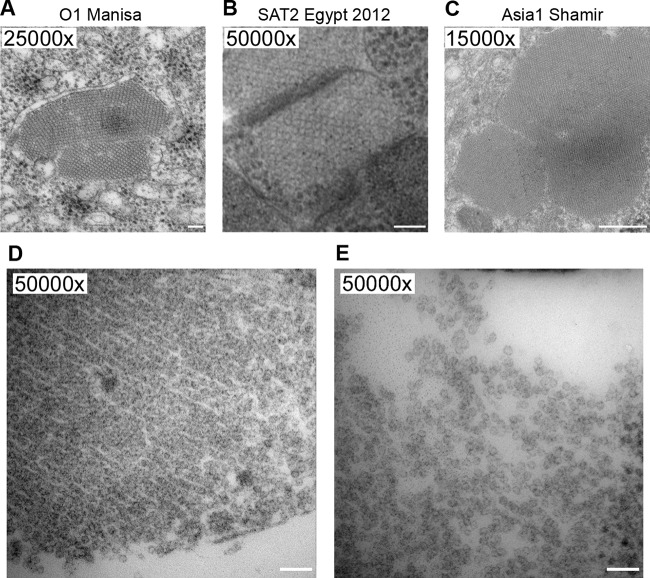
(A to C) TEM images of FMDV VLP arrays in HEK293-T cells transfected with plasmids encoding 3C(L127P) and P1 polyprotein from (A) O1 Manisa, (B) SAT2 Egypt 2012, or (C) Asia1 Shamir. (D and E) FMDV O1 Manisa VLPs emerging from transfected HEK293-T cells (D) and dispersing into individual capsids (E). Bars in panels A and B and on the right side of panels D and E, 100 nm; bar in panel C, 500 nm.

When produced at high concentrations, assembled FMDV capsids and VLPs can be observed as crystalline arrays within cells by transmission electron microscopy (TEM) (22, 26). In this study, crystalline arrays were seen in mammalian cells transfected with P1-3C(L127P) constructs encoding P1 polyproteins from FMDV serotypes O, Asia1, and SAT2 (Fig. 3A to C). Cells expressing FMDV serotype O P1-3C(L127P) were observed producing crystalline arrays in the cytoplasm which disassociated at the plasma membrane (Fig. 3D) to release individual VLPs from the cell (Fig. 3E). Although crystalline arrays were not observed for all FMDV serotypes tested, previous TEM studies indicated that crystalline arrays of VLPs are not always found for subunit vaccine constructs that are efficacious in cattle trials (unpublished data).

### Effect of 3C(L127P) on mammalian host proteins.

**FIG 4:**
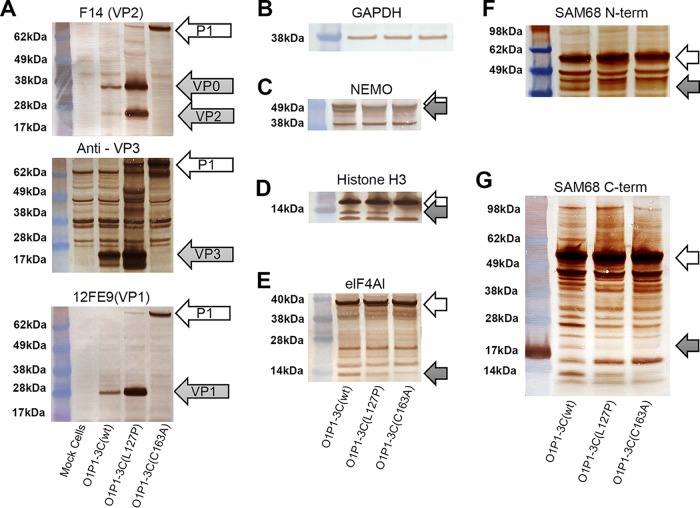
Western blots of lysates from HEK293-T cells transfected with FMDV O1 Manisa P1 and 3C variants (wild-type strain or L127P or C163A mutant). White arrows indicate unprocessed host protein. Gray arrows indicate processed fragments. (A) Western blots showing FMDV P1 polyprotein and processed products of VP0/VP2, VP3, and VP1. (B) Equal levels of loading indicated by anti-GAPDH antibody analysis. (C to G) Blots of 3C protease host protein targets: (C) anti-NEMO; (D) anti-histone H3; (E) anti-eIF4AI; (F) anti-SAM68 N terminus (N-term); (G) anti-SAM68 C terminus (C-term).

To further characterize the mechanism behind the increased yields of recombinant proteins in mammalian cells associated with 3C(L127P), HEK293-T cells were transfected with plasmids encoding the FMDV O1 Manisa P1 polyprotein and a 3C variant (wt, L127P, or C163A). Cell lysates were analyzed for cleavage products from FMDV and host proteins with equal levels of loading of cell lysates determined by blotting for GAPDH (glyceraldehyde-3-phosphate dehydrogenase) (Fig. 4B). Western blots showed higher yields of FMDV VPs in cells transfected with O1P1-3C(L127P) than in those transfected with O1P1-3C(wt) (Fig. 4A), confirming the luciferase assay results. Cells transfected with O1P1-3C(C163A) had no detectable processed P1 polyprotein, confirming the knockout of proteolytic activity (Fig. 4A).

Degradation of host proteins was evaluated by Western blotting for four known host protein targets: NEMO, histone H3, SAM68, and eIF4AI. Lysates from HEK293-T cells transfected with O1P1-3C(C163A), the negative control, showed no detectable degradation of these four host proteins (Fig. 4C to G). Lysates from cells transfected with O1P1-3C(wt) showed degradation products for all four tested host proteins. The 3C(L127P) mutation completely blocked degradation of NEMO (Fig. 4C) and SAM68 (Fig. 4F and G) and reduced degradation of eIF4AI (Fig. 4E) and histone H3 (Fig. 4D).

SAM68 is degraded in FMDV-infected cells, resulting in a 40-kDa N-terminal fragment and intracellular redistribution of the protein (15). In this study, lack of a 40-kDa band indicated that 3C(L127P) did not cleave SAM68 (Fig. 4F). Western blots using an antibody to the C terminus of SAM68 showed a unique band at around 20 kDa in the O1P1-3C(wt) sample only, indicating another possible degradation product of SAM68 resulting from 3C(wt) but not from the 3C(L127P) variant or the 3C(C163A) variant (Fig. 4G).

**FIG 5:**
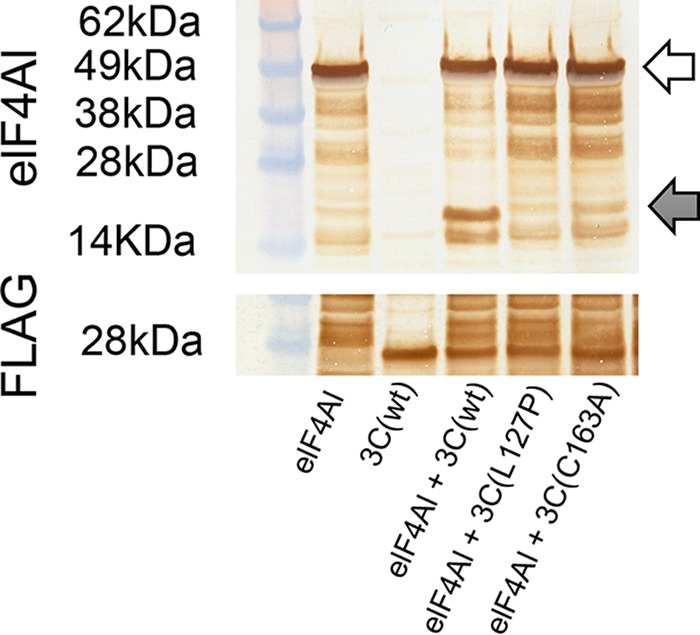
Western blot analysis of cell-free reactions with anti-eIF4AI and anti-FLAG antibodies. White arrows indicate unprocessed host protein. Gray arrows indicate processed fragments.

Degradation of eIF4AI by 3C(wt) shuts down host translation in mammalian cells (13), and the 3C(L127P) mutant degraded eIF4AI less than 3C(wt) (Fig. 4E). To further characterize the effect of 3C(L127P) on bovine eIF4AI under uniform expression conditions, a pSNAP plasmid encoding bovine eIF4AI was coexpressed in a cell-free system at equimolar concentrations with a second pSNAP plasmid encoding 3C(L127P), 3C(C163A), or 3C(wt). Results showed that degradation of bovine eIF4AI by 3C(L127P) and 3C(C163A) was negligible compared to that by 3C(wt) (Fig. 5).

### Use of 3C(L127P) in an adenovirus serotype 5 (Ad5) vaccine vector.

**FIG 6:**
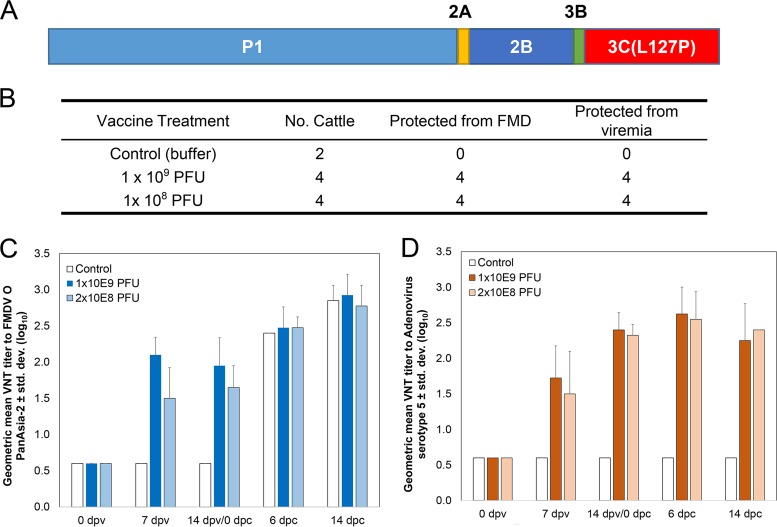
Data from vaccination and challenge study with cattle. (A) Transgene layout of FMDV serotype O PanAsia-2 sequence cloned into Ad5 Blue vector. (B) Number of cattle protected from clinical FMD and viremia by treatment group at 3, 6, 10, and 14 dpc. *P* = 0.07 (for comparisons of each vaccinated group to the control group; Fisher's exact test). All cattle were challenged 14 dpv with virulent FMDV serotype O PanAsia-2. (C) FMDV serotype O PanAsia-2 virus neutralization test antibody titers (VNT) in serum samples. (D) VNT titers to adenovirus serotype 5 vector. For panels C and D, bars represent the VNT geometric mean titer (GMT) for 4 values (± standard deviations; limit of detection range, >0.6 to 3.6 log_10_). dpv, days postvaccination; dpc, days postchallenge.

To evaluate use of 3C(L127P) in a molecular subunit vaccine, the replication-deficient recombinant adenovirus vector pAd5-Blue backbone (27) was used to encode a FMDV P1 polyprotein (serotype O PanAsia-2), 2A, 2B protein, 3B protein, and 3C(L127P) protease (Fig. 6A), similarly to previously tested Ad-FMD vaccines containing 3C(wt) (3, 28, 29). All eight cattle immunized with a single dose of 3C(L127P)-containing vaccine were protected from clinical FMD and viremia following challenge with homologous FMDV serotype O PanAsia-2 at 14 days postvaccination (dpv) (*P* = 0.07 [Fisher's exact test]) (Fig. 6B). All vaccinated cattle produced serum neutralizing antibodies to both FMDV O PanAsia-2 and the adenovirus serotype 5 vector at 7 and 14 dpv (Fig. 6C and D). Clinical protection and serum neutralizing antibody titers were consistent with previous results obtained using other Ad-FMD vaccines (2, 3).

### Effect of expression of the 3C mutants in Escherichia coli.

**FIG 7:**
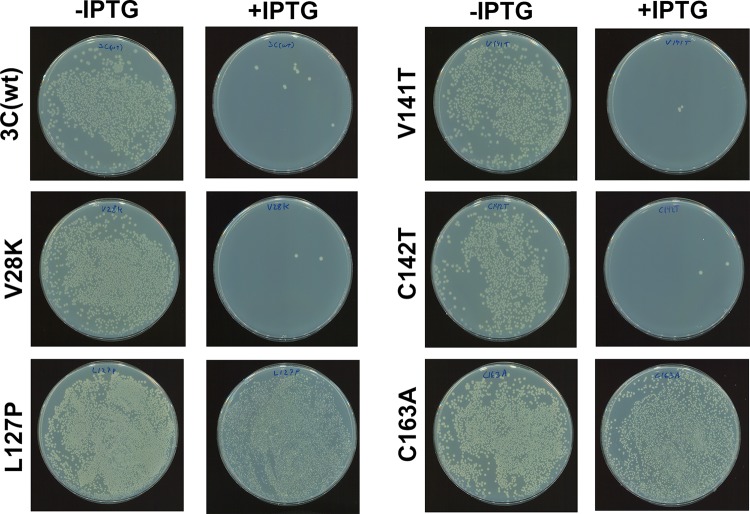
Overnight growth of E. coli bacteria expressing 3C mutants ± the inducer, IPTG.

We evaluated the ability of the 3C(L127P) mutant to enable bacterial expression as a practical platform for which 3C(wt) toxicity had previously been problematic. When 3C protease mutants were expressed in E. coli under conditions of Lac I regulation, approximately 1,000 more colonies expressing the 3C(C163A) or 3C(L127P) mutants survived after overnight induction (IPTG [isopropyl-β-d-thiogalactopyranoside]) compared to E. coli transfected with the other three mutants or the wild-type 3C gene (Fig. 7), demonstrating that the 3C(L127P) mutation also mitigated the detrimental effects of 3C expression in the bacterial system.

**FIG 8:**
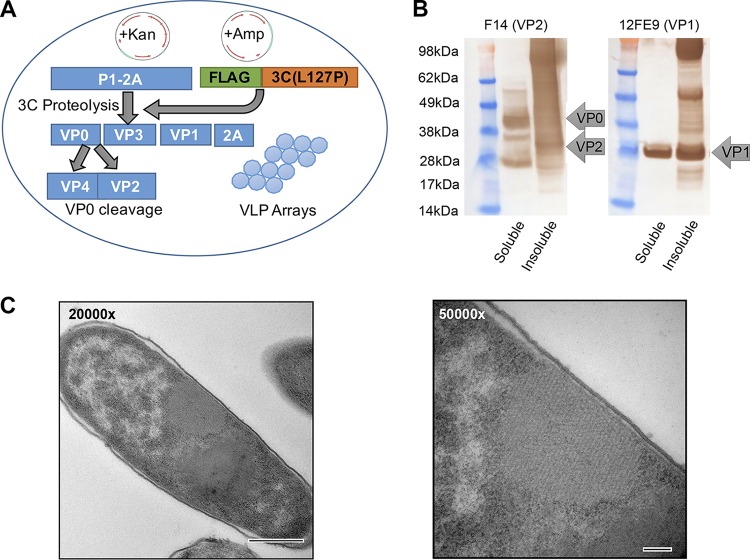
(A) Diagram of the two-plasmid system used for expression of FMDV P1 and 3C protease in E. coli to produce virus-like particles. (B) Western blot analyses of soluble and insoluble fractions from cell lysates of induced E. coli expressing FMDV O1 Manisa P1 and FLAG-tagged 3C(L127P) demonstrating the presence of processed VP0, VP2, and VP1. (C) TEM image of VLP crystalline arrays in transformed bacteria expressing FMDV O1 Manisa P1 and 3C(L127P). Bar on 20000× panel, 500 nm; bar on 50000× panel, 100 nm.

Coexpression of FLAG-tagged 3C(L127P) and P1-2A proteins in E. coli (Fig. 8A) resulted in fully processed FMDV O1 Manisa P1, as determined by Western blotting detection of VP0, VP2, and VP1, in both the soluble and insoluble fractions (Fig. 8B). Cotransformation of E. coli with FMDV O1 Manisa P1 and 3C(L127P) genes also produced crystalline arrays of FMDV VLPs (Fig. 8C), as seen by TEM.

## DISCUSSION

The results of this study show that the FMDV 3C(L127P) mutant significantly increased yields of recombinant FMDV capsid proteins compared to wild-type 3C protease and produced FMDV VLPs in both mammalian and bacterial cells. Furthermore, cattle immunized with a single dose of Ad5-FMD P1-3C(L127P) vaccine were protected from clinical disease after challenge with homologous FMDV.

The increased yields of recombinant vaccine antigens associated with use of 3C(L127P) appear to result from reduced degradation of host proteins allowing better expression of transgenes in transformed cells. The L127 residue resides on the B_2_ β-strand in published crystal structures (30, 31). It has been previously hypothesized that the nearby A_2_-B_2_ loop in the FMDV 3C protease may contribute to substrate specificity (30). The results showing reduced cleavage of host proteins NEMO, SAM68, histone H3, and eIF4AI by 3C(L127P) collectively would support the conclusion that this region of the 3C protease can play a critical role in substrate specificity despite being distal to the active site. In particular, reduced degradation of eIF4AI by 3C(L127P) indicates less inhibition of host cell translation than was seen with 3C(wt). Shutdown of this pathway by 3C(wt) has been a hindrance for multiple molecular vaccine platforms, particularly those which utilize cap-dependent translation for antigen expression. NEMO inhibits nuclear factor kappa-B kinase subunit gamma and supports type I interferon production (16). The lack of NEMO degradation by 3C(L127P) compared to 3C(wt) might mitigate this viral immune evasion mechanism and might possibly enhance the efficacy of recombinant FMD vaccines containing a 3C(L127P) protease gene.

A reduction in toxic effects on host cells by 3C(L127P) should also improve production yields of recombinant subunit FMD vaccines and vaccine potency. For example, current methods of adenovirus-vectored FMDV vaccine production seek to reduce or eliminate expression of 3C(wt) in order to limit toxicity to host cells during the manufacturing process. The reduced toxicity associated with the 3C(L127P) protease may improve manufacturing yields by limiting toxicity in the system or may lower costs by eliminating the inhibitors currently required during the production cycle. Moreover, vaccination with Ad-FMD vectors using the 3C(L127P) protease may have benefits with respect to intrinsic antigen expression. In this study, cattle immunized with a single dose of Ad5-FMD P1-3C(L127P) vaccine were protected from clinical disease after challenge with homologous FMDV. This proof-of-concept clinical study demonstrated the efficacy of the 3C(L127P) protease in a well-characterized, successful FMDV vaccine vector platform. The Ad-FMD vaccine results support further evaluation of the use of the 3C(L127P) protease in experimental Ad5-FMD subunit vaccines currently in development.

The most striking result of using 3C(L127P) was the clear demonstration of abundant intact FMDV VLPs produced in E. coli indicated by intracellular crystalline arrays as seen by electron microscopy. To our knowledge, this is the first demonstration of FMDV VLP crystalline array structures forming in a bacterial cell. Crystalline array structures were not seen in E. coli transfected with FMD vaccine constructs expressing 3C(wt) (unpublished results). Incorporation of the 3C(L127P) protease into FMD vaccine constructs may enable bacterial expression of FMD subunit vaccines as a safe and economical production method that could significantly reduce costs compared to FMD vaccine production in eukaryotic cells. As such, the 3C(L127P) mutant advances the potential to use bacterial expression as a production platform for recombinant FMD subunit vaccines, a long-sought goal of the FMD vaccine community.

## MATERIALS AND METHODS

### Preparation of pTarget GLuc-Δ1D2A-3C constructs.

The pTarget GLuc-Δ1D2A plasmid vector was constructed as previously described (22). Amplification of the 3C protease gene from an FMDV Asia1 Lebanon 1989 (GenBank accession no. AY593798) noninfectious template was performed using OneTaq 2× master mix with Standard Buffer (New England BioLabs) and primers XmaI-3C-F (CTACCCGGGCCGAGTGGTGCCCCAC) and 3C-NotI-R (TAGCGGCCGCTACTCGTGGTGTGGTTC). The PCR product was purified using a PCR purification kit (Qiagen). Both the PCR product and pTarget GLuc-Δ1D2A vector were digested with XmaI and NotI-HF restriction enzymes (New England BioLabs). Ligations were performed using T4 DNA ligase (Roche) and transformed into NEB 5-alpha competent E. coli (New England BioLabs). Plasmids were isolated using a QIAprep Spin Mini-prep kit (Qiagen) and were amplified with primers T7 (TAATACGACTCACTATAGGG) and Seq-R (TTACGCCAAGTTATTTAGGTGACA) for sequencing, and results were analyzed with Sequencher 4.8 software (Genecodes).

### Construction of V28K, L127P, V141T, and C163A mutants.

The V28K, V141T, and C163A mutants were produced by using a GeneArt site-directed mutagenesis system (Invitrogen) with the following primer sets for the respective mutations: 3CLeb89 V28K-MF (CTTGACGGTAAGACGAAGGCCATCTGCTGCGC) and 3CLeb89 V28K-MR (GCGCAGCAGATGGCCTTCGTCTTACCGTCAAG), 3CLeb89 V141T-MF (TACAAGGACATTGTAACGTGCATGGATGGAGA) and 3CLeb89 V141T-MR (TCTCCATCCATGCACGTTACAATGTCCTTGTA), and 3C C163A-MF (ACCAAGGCTGGCTACGCTGGAGGAGCCGTTCT) and 3C C163A-MR (AGAACGGCTCCTCCAGCGTAGCCAGCCTTGGT). Site-directed mutagenesis was performed using 45 μl of Accuprime *Pfu* Supermix (Life Technologies), 5 μl of 10× enhancer (Invitrogen), 1 μl of DNA methylase (Invitrogen), 0.25 μl of 200× SAM (Invitrogen), 0.1 μl of pTarget GLuc-Δ1D2A-3C, and 250 ng of each primer with the following thermocycling profile: 37°C for 20 min; 94°C for 2 min; 17 cycles of 94°C for 20 s, 57°C for 30 s, and 68°C for 3 min 30 s; and 68°C for 5 min. The recombination reaction and E. coli transformation were performed as recommended by the manufacturer. Sequencing and analysis were performed as described above using primers T7 and Seq-R. The L127P mutant was produced by PCR polymerase infidelity during the cloning of wild-type 3C.

### Construction of pTarget GLuc-Δ1D2A-HIV-3C(C142T) plasmid.

The pTarget GLuc-Δ1D2A-HIV-3C(C142T) plasmid was constructed based on the previously described pTarget GLuc-Δ1D2A plasmid (22), with the sequence for HIV-3C(C142T) derived as described in a previous publication (9), synthesized by GenScript, and inserted using XmaI and NotI restriction enzymes as described above.

### Transfection.

HEK293-T cells (ATCC CRL-3216), passage 41, were grown on Costar 6-well plates (Corning Incorporated) in 293 growth media (1× minimal essential medium [MEM], 10% fetal bovine serum, 1% 100× GlutaMAX, 1% MEM nonessential amino acids, and 1% antibiotic-antimycotic solution). Cells at roughly 80% confluence were rinsed with 2 ml of 1× Dulbecco's phosphate-buffered saline (DPBS) (Gibco), and 2 ml of fresh medium was added to each well. Transfections were performed using 4 μg of plasmid DNA and 10 μl of Lipofectamine 2000. Transfected cells were placed in a 37°C CO_2_ incubator for 24 h for protein expression.

### Luciferase assay.

Luciferase activity in harvested cell culture medium was measured using a 96-well BioSystems Veritas luminometer (Turner Biosystems) with 20 μl of sample in each well. Luminescence output was measured immediately upon injection of 25 μl of 50 μg/μl coelenterazine solution (NanoLight Technologies, Pinetop, AZ) using an integration time of 0.5 s both before and after injection of substrate. Preinjection readings were used to establish a baseline at the time of injection and were subtracted from postinjection values for data analysis. Replicate data were averaged together to give the overall luciferase activity in relative luciferase units per half-second (RLU/0.5 s). Six or seven replicates were used for each sample (see figure legends). Data were analyzed initially by single-factor analysis of variance (ANOVA), which indicated significance. The two-tailed *t* test (with unequal levels of variance) was then used to compare luciferase RLU/0.5 s data between one construct and another. A *post hoc* one-tailed *t* test analysis was used to determine whether the luciferase RLU/0.5 s data from the 3C(L127P) constructs were different from the data determined for the other 3C mutants and wild-type constructs.

### Construction of pSNAP FLAG-3C and pET O1P1-2A plasmids.

The FLAG-3C(wt) gene was synthesized by GenScript and inserted into pSNAP-tag (T7)-2 vector (New England BioLabs) using restriction enzymes NdeI and XhoI as described above. To produce V28K, V141T, and C163A mutants, site-directed mutagenesis was performed using the primers and protocol described above. To produce L127P and C142T mutants, site-directed mutagenesis was performed as described above using the following primer sets: 3C L127P-MF (CCGACGTTGGGAGACCGATTTTCTCCGGTGA) and 3C L127P-MR (TCACCGGAGAAAATCGGTCTCCCAACGTCGG) as well as 3C C142T-MF (AAGGACATTGTAGTGACCATGGATGGAGACAC) and 3C C142T-MR (GTGTCTCCATCCATGGTCACTACAATGTCCTT), respectively. For construction of the pET O1P1-2A plasmid, a NotI restriction site was inserted into a Champion pET SUMO expression system (Thermo Fisher). The FMDV O1 Manisa P1-2A polypeptide gene was synthesized (GenScript) with flanking NheI and NotI restriction sites. The NheI and NotI restriction sites were used to insert the P1-2A gene into the modified pET SUMO plasmid, replacing the SUMO sequence.

### Transformation of pSNAP FLAG-3C constructs.

To evaluate the effect of 3C expression in bacteria, pSNAP FLAG-3C plasmids were transformed into T7 Express competent E. coli (New England BioLabs) and incubated in 10 ml of 100 μg/ml carbenicillin Terrific broth on a shaker for 3 h at 37°C. After incubation, viable E. coli cells were quantified using the BacTiter-Glo microbial cell viability assay (Promega). For all samples, equivalent numbers of viable E. coli cells were mixed in 200 μl containing 100 μg/ml carbenicillin in Terrific broth, and 75 μl was plated on LB plates containing either 100 μg/ml carbenicillin or 100 μg/ml carbenicillin, 0.1 mM IPTG, and 60 μg/ml X-Gal (5-bromo-4-chloro-3-indolyl-β-d-galactopyranoside). Plates were incubated at 37°C overnight and examined for growth.

### Expression and Western blot analysis of pSNAP FLAG-3C and pET O1P1-2A transformed E. coli.

For expression in bacteria, the pSNAP FLAG-3C and pET O1P1-2A plasmids were transformed into T7 Express competent E. coli (New England BioLabs) and grown in LB broth containing 100 μg/ml carbenicillin and 50 μg/ml kanamycin (Teknova) overnight at 37°C. Cultures were split, and an equal amount of fresh media was added. To induce expression, IPTG was added to reach a final concentration of 1 mM, and cultures were incubated on a shaker overnight at 30°C.

Induced bacterial cultures were pelleted by centrifugation at 6,800 × *g* for 3 min. Supernatant was removed and the pellet dissolved in 500 μl of B-PER bacterial protein extraction reagent (Thermo). After the resuspended bacteria were subjected to one freeze-thaw cycle, 5 μl of DNase I (New England BioLabs) was added, and the treated bacteria were incubated at 37°C for 1 h. Protein expression and processing were evaluated by Western blotting using precast polyacrylamide (4% to 12%) NuPAGE Bis-Tris gels transferred to a 0.2-μm-pore-size polyvinylidene difluoride (PVDF) membrane and probed with F1412SA (anti-VP0 and anti-VP2) mouse monoclonal antibody at a 1:50 dilution (32) and 12FE9.2.1 (anti-VP1) mouse monoclonal antibody at a 1:50 dilution (33); both dilutions were performed using PBS with Tween 20 (PBS-T). Blots were blocked in 5% milk for 1 h, followed by three 5-min washes performed with 1× PBS-T buffer. Primary antibodies were incubated for 1 h at room temperature, and membranes were washed three times with 1× PBS-T buffer for 5 min each time. Secondary antibody of goat anti-mouse horseradish peroxidase (HRP) (KPL) was applied to membranes for 1 h at room temperature followed by three 5-min washes with 1× PBS-T. Blots were developed with SigmaFAST 3,3′-diaminobenzidine (Sigma) for 1 h at room temperature or until bands were clearly visible followed by two washes with double-distilled water (ddH_2_O).

### Construction of P1-2A-3C-SGLuc plasmids.

The P1-2A-3C-SGLuc plasmids were constructed using a modified pTarget (mpTarget) vector (Promega). Modifications included deletions to decrease the overall vector size, removal of the multiple-cloning site (MCS) 5′ EcoRI cut site, and addition of an XmaI cut site on the 3′ end of the MCS. Nucleotide sequences for P1-2A were synthesized by GenScript and derived from O1 Manisa (GenBank accession no. AY593823), A24 Cruzeiro (GenBank accession no. AY593768), Asia1 Shamir (GenBank accession no. JF739177), C3 Indaial (GenBank accession no. AY593806), SAT1 KNP/196/91 (GenBank accession no. DQ009716), SAT2 Egypt 2012 (GenBank accession no. KC440884), and SAT3 ZIM/05/91 (GenBank accession no. DQ009740). Synthesized genes were inserted into the mpTarget vector using cut sites BamHI and NotI. Ligation, cloning, and sequencing were performed as described above.

Amplification of 3C variant genes from pTarget GLuc-Δ1D2A-3C wild-type, L127P, and C163A constructs was performed using OneTaq 2× master mix with Standard Buffer (New England BioLabs) and primers NotI-3CLeb89-F (CAGCGGCCGCATGAGTGGTGCCCCACCG) and 3C Asia-ns-EcoRI-R (GAATTCCTCGTGGTGTGGTTC). PCR product was purified using a PCR purification kit (Qiagen). Both the PCR product and mpTarget P1-2A plasmids were digested with NotI-HF and EcoRI-HF restriction enzymes (New England BioLabs). Ligation, cloning, and sequencing were performed as described above.

The Δ1D2A-SGLuc gene was commercially synthesized (GenScript) and digested with restriction enzymes EcoRI-HF and XmaI (New England BioLabs) along with the mpTarget P1-2A-3C(L127P) template vectors. Ligation, cloning, and sequencing were performed as described above.

### Transfection and harvesting of mammalian cells transfected with P1-2A-3C-SGLuc constructs.

We transfected HEK293-T cells with mpTarget P1-2A-3C(L127P)-SGLuc and quantified luciferase activity as previously described for pTarget GLuc-Δ1D2A-3C constructs (22). Cells were harvested by resuspension in fresh growth media and centrifugation at 6,800 × *g* for 5 min to pellet cells. Cells were resuspended in 200 μl of M-PER mammalian protein extraction reagent (Invitrogen). Samples were mixed with 4× NuPAGE loading buffer (Invitrogen) and heated at 95°C for 10 min before centrifugation and loading onto NuPAGE Novex 4% to 12% Bis-Tris protein gels (Invitrogen) and run in 1× MES Buffer at 200 V for 35 min. Transfer to membranes was performed using 0.2-μm-pore-size PVDF precut blotting membranes (Invitrogen) with 1× Transfer Buffer (Invitrogen).

### Western blot analysis of mpTarget P1-2A-3C(L127P)-SGLuc constructs.

Loading of cell lysate samples was normalized to luciferase readings from media harvested from mpTarget P1-2A-3C(L127P)-SGLuc transformations. Western blot analysis was performed to investigate the presence of processed VPs using F1412SA (anti-VP0 and VP2) mouse monoclonal antibody at a 1:50 dilution (32), an anti-VP3 rabbit polyclonal antibody at a 1:250 dilution (made by us), 6HC4.1.3 (anti-VP1) mouse monoclonal antibody at a 1:50 dilution (34), and 12FE9.2.1 (anti-VP1) mouse monoclonal antibody at a 1:50 dilution (33); all dilutions were in PBS-T. Blots were blocked in 5% milk for 1 h, followed by three 5-min washes with 1× PBS-T buffer. Blots were incubated with primary antibodies for 1 h at room temperature, and then membranes were washed three times for 5 min each time with 1× PBS-T buffer. Secondary antibodies, either goat anti-mouse HRP antibody (KPL) or goat anti-rabbit HRP antibody (KPL) at a 1:500 dilutions, were applied to membranes for 1 h at room temperature followed by three 5-min washes with 1× PBS-T. Blots were developed with SigmaFAST 3,3′-diaminobenzidine (Sigma) for 1 h at room temperature followed by two washes with ddH_2_O. In order to detect VP2 in SAT2 samples, we used a nonnormalized blot with an increased amount of the L127P cell lysate.

### Transmission electron microscopy (TEM).

For TEM of cell cultures, transfected HEK293-T cells were grown in T-75 flasks and transfected with mpTarget P1-2A-3C(L127P)-SGLuc constructs. Transfected HEK293-T cells were incubated overnight at 37°C in 5% CO_2_ prior to processing. Transformed bacterial cells were induced, cultured, and pelleted as described above prior to processing. For TEM processing, cells were fixed in 2% glutaraldehyde–NaHCa (Heuser's) buffer, postfixed with 1% tannic acid followed by 1% osmium tetroxide, stained *en bloc* with 4% uranyl acetate, embedded in 2% agarose, dehydrated through a graded series of acetone, and embedded in Spurr's resin (Electron Microscopy Sciences). Ultrathin (80-nm) sections were cut on a Leica UC6 microtome, stained with uranyl acetate and lead citrate, and imaged on a Hitachi 7600 analyzer with a 2-k-by-2-k AMT camera at 80 kV.

### Transformation and Western blotting of host cell proteins.

Transformation of HEK293-T cells with mpTarget O1P1-3C(L127P), mpTarget O1P1-3C(wt), or mpTarget O1P1-3C(C163A) constructs was performed as described above. Equivalent levels of loading of lysate proteins were determined with anti-GAPDH antibody (ab9385; Abcam). Western blots for FMDV VPs using F1412SA, anti-VP3, and 12FE9.2.1 antibodies was performed as described above. Western blots of host proteins used the following antibodies as described above: anti-IκB kinase (anti-IKK) gamma (ab137363; Abcam) for NEMO, anti-histone H3 (ab1791; Abcam), anti-eIF4AI (ab31217; Abcam), and anti-SAM68 (ab86239 for N terminus and ab26803 for C terminus; Abcam).

### Cell-free protein synthesis and Western blotting.

The bovine eIF4AI gene (GenBank accession no. 77735406) was synthesized by GenScript with the addition of an N-terminal FLAG tag and a C-terminal myc tag and cloned into pSNAP-tag (T7)-2 vector (New England BioLabs) using cut sites NdeI and NotI. Nucleotide sequences for 3C(wt), 3C(L127P), and 3C(C163A) with N-terminal FLAG tags were also cloned into pSNAP-tag (T7)-2 vector (New England BioLabs) using cut sites NdeI and NotI. Cell-free protein synthesis used a PURExpress *in vitro* protein synthesis kit (New England BioLabs) with the modification that two DNA plasmids were added in equimolar amounts. Western blots of cell-free synthesis products used anti-eIF4AI (ab31217, Abcam) antibody to detect eIF4AI and anti-DYKDDDDK (635691; TaKaRa) antibody to detect FLAG-tagged 3C.

### Ad5 Blue vector construction.

The P1-2A-2B-3B-3C(L127P) gene construct encoding the FMDV serotype O PanAsia-2 (O/IBD/PAK/10/2010) sequence with flanking ClaI and XbaI restriction enzyme sites was synthesized by GenScript. The Ad-FMD vaccine was constructed on an Ad5 Blue vector backbone as previously described (27).

### Vaccination and challenge.

Prior to conducting the cattle study, prior approval was obtained from the Plum Island Animal Disease Center Institutional Review Board and the Institutional Animal Care and Use Committee. Ten Holstein heifers, 6 to 7 months old, were randomly assigned to three groups as follows: group 1, buffer control cattle (*n* = 2); group 2, 1 × 10^9^ PFU vaccine dose cattle (*n* = 4); group 3, 1 × 10^8^ PFU vaccine dose cattle (*n* = 4). Both the experimental vaccines and the control vehicle (final formulation buffer [Lonza]) were formulated with ENABL C1 adjuvant (VaxLiant). Cattle were challenged intradermolingually with 10^4^ 50% bovine infectious doses of FMDV O PanAsia-2 at 14 days postvaccination (dpv). Clinical FMD (pedal and oronasal epithelial lesions) was assessed by visual inspection at 3, 6, 10, and 14 days postchallenge (dpc). Serum samples collected at 0, 7, and 14 dpv and at 6 and 14 dpc were evaluated for virus neutralizing test (VNT) antibody titers against FMDV O PanAsia-2 and human adenovirus serotype 5 (Ad5) by serial dilution of heat-inactivated serum samples. VNT antibody titers were determined by a constant virus, decreasing serum microneutralization test. VNT titers were measured in BHK-21 cell cultures by the use of 100 to 150 50% tissue culture infective doses (TCID_50_) of FMDV serotype O PanAsia/IBD-10/ARS-PAK/2010 and in HEK293 cells using 250 TCID_50_ of Ad5. VNT titers were calculated by using the Spearman-Kärber method based on cytopathic effect (CPE). VNT titers of ≥0.9 log_10_ were scored as positive (3). Plasma samples were collected at 0 to 5 dpc and were evaluated for viremia by real-time reverse transcription-PCR (rRT-PCR) (A positive *C_T_* [threshold cycle] value was ≤40) and FMDV isolation on LFBK-αvβ6 cells (3). Protection from clinical FMD or viremia was analyzed statistically with Fisher's exact test.
